# Smart Sensors for Augmented Electrical Experiments

**DOI:** 10.3390/s22010256

**Published:** 2021-12-30

**Authors:** Sebastian Kapp, Frederik Lauer, Fabian Beil, Carl C. Rheinländer, Norbert Wehn, Jochen Kuhn

**Affiliations:** 1 Physics Education Research Group, Department of Physics, Technische Universität Kaiserslautern, 67663 Kaiserslautern, Germany; beil@physik.uni-kl.de (F.B.); kuhn@physik.uni-kl.de (J.K.); 2 Microelectronic Systems Design Research Group, Department of Electrical and Computer Engineering, Technische Universität Kaiserslautern, 67663 Kaiserslautern, Germany; flauer@eit.uni-kl.de (F.L.); rheinlaender@eit.uni-kl.de (C.C.R.); wehn@eit.uni-kl.de (N.W.)

**Keywords:** augmented reality, smart sensors, STEM education, laboratory instruction

## Abstract

With the recent increase in the use of augmented reality (AR) in educational laboratory settings, there is a need for new intelligent sensor systems capturing all aspects of the real environment. We present a smart sensor system meeting these requirements for STEM (science, technology, engineering, and mathematics) experiments in electrical circuits. The system consists of custom experiment boxes and cables combined with an application for the Microsoft HoloLens 2, which creates an AR experiment environment. The boxes combine sensors for measuring the electrical voltage and current at the integrated electrical components as well as a reconstruction of the currently constructed electrical circuit and the position of the sensor box on a table. Combing these data, the AR application visualizes the measurement data spatially and temporally coherent to the real experiment boxes, thus fulfilling demands derived from traditional multimedia learning theory. Following an evaluation of the accuracy and precision of the presented sensors, the usability of the system was evaluated with n=20 pupils in a German high school. In this evaluation, the usability of the system was rated with a system usability score of 94 out of 100.

## 1. Introduction

In the context of STEM (science, technology, engineering, and mathematics) education, it is essential to teach students not only the facts but also the ways of thinking about various disciplines and conceptual connections (e.g., [1,2]). This is often accomplished through the use of inquiry learning, in which students use experiments and research processes to construct knowledge [3]. Although traditional physical and hands-on laboratory experiments targeted at inquiry learning provide unique experiences, pure physical laboratories do not necessarily result in learning success [4,5,6,7,8]. Specific guidance and appropriate educational support to build methodological knowledge, however, can play an essential role in increasing learning outcomes [9,10]. Otherwise, situations of cognitive overload due to the complexity of the processes can be experienced by learners, hindering their learning (e.g., [11]).

To counteract this problem, classical hands-on experiments have previously been enriched using virtual supporting information [10,12,13]. One approach, for example, consists of supplementing traditional physics experiments with additional virtual representations [13,14]. Here, the use of augmented reality (AR) to present additional external representations increasingly comes into focus in educational research [15,16,17,18]. Using AR in laboratory settings thereby enables the integration of virtual information (e.g., measurement data) into the real environment without hindering interaction [18,19,20]. This allows formerly invisible phenomena and abstract quantities to become visible (e.g., heat or electricity) as well as visualizing functional relations between real components and virtual visualizations using the spatial and temporal contiguity between them [12,21]. These possibilities have resulted in increasing use of AR in STEM education [18,22,23,24].

The biggest challenge in using AR in STEM education is merging the virtual world with physical reality. Therefore, advanced sensor systems that can capture as many variables of the experiment as possible are essential to enable seamless integration [25]. Simultaneously, this wealth of real-time information regarding the current state of the experiment offers tremendous opportunities for educational data mining and AI-based, individualized feedback, or other data-driven applications in the emerging field of technology-enhanced learning [26,27,28,29].

In this paper, we present a new system based on smart sensors integrated into electrical experiment components for inquiry-based learning in STEM disciplines that can be seamlessly integrated into AR environments for visualization and, in the future, for diagnosis and AI applications. In particular, we consider the various integrated sensor systems which, in addition to the seamless integration of the virtual elements into the physical environment, also enable the creation of an accurate digital twin. Following the core functions of smart learning environments identified by Tabuenca et al. [30], we focus on three facets in particular:

- Sense: Our system is capable of collecting information from the context in which it is introduced;
- Analyze: Our system is able to generate higher-level indicators from the data collected in the sensing process using data analysis techniques;
- React: While AR has typically been employed without sensor input or cumbersome, separate sensors, our system enables opportunities for new and expanded experiments through integrated sensors, such as educational data mining analysis and AI-based, individualized feedback using the same sensor technology. In this way, our system will prospectively provide customized recommendations for stakeholders based on the data collected during the sensing process and its interpretation performed during the analysis process.

The key contributions of this paper are as follows:

- A smart sensor system for educational STEM experiments in electrical circuits consisting of sensors for voltage and current measurement, position identification with a focus on a 2D plane, and cable identification for circuit reconstruction;
- Energy-efficient and robust data transmission of the measured values to the Microsoft HoloLens 2 through the use of the widely used Bluetooth Low Energy (BLE) standard;
- Software solution for processing and visualizing sensor values in AR on the Microsoft HoloLens 2;
- An evaluation of the installed sensor systems in terms of accuracy and precision;
- An initial field study with students to evaluate the usability of the whole system consisting of multiple sensor boxes and including the first AR application running on the Microsoft HoloLens 2.

Bringing together interdisciplinary groups of disciplinary education, cognitive science, HCI, and engineering, we fulfill technological and educational requirements as well as design principles derived from established theories of multimedia information processing.

The paper is structured as follows: Section 2 covers the related work regarding cognitive load theory and the cognitive theory of multimedia learning as well as the topic of sensors for AR. In Section 3, we present a detailed description of the sensors integrated in our system. The transmission and processing of the sensor values are covered in Section 4. Section 5 describes the results of the evaluations performed with respect to the accuracy and precision of the sensors as well as the usability of the overall system. The discussion in Section 6 and the conclusion in Section 7 sum up the results, discuss current limitations, and provide concepts for the further development of the system.

## 2. Background and Related Work

In this section, we derive the requirements for our new sensor system from existing theories regarding the cognitive load during learning with multiple external representations (MER) and sensors for AR in general. Additionally, we provide a short overview of the SteamVR locating system which we have adapted to form our new position identification system.

### 2.1. Learning with Multiple External Representations (MER)

The combined presentation of virtual visualizations with real components in STEM experiments can, in general, be understood as MERs. They give access to to-be-learned information as the virtual elements can be composed of measuring data, text, symbolic elements, or schematized representations of physical objects as well as representations of abstract elements. The importance of multiple representations during STEM learning has been widely documented [31], especially in physics [32,33]. In this context, they represent an essential part of the exchange of information and form a central element in the development of conceptual understanding [34,35,36,37]. To describe the structure and functions of MERs, Ainsworth [38,39] created the conceptual DeFT framework providing an overview of the prerequisites for their effective use. The authors describe MERs in the context of learning scenarios according to the aspects of design, function, and task. In Ainsworth [38], the authors thereby identified three core functions of MERs that promote learning: (a) they can provide different and complementary information or allow complementary approaches to process information, (b) they mutually influence the use or interpretation of the representations involved through familiarity with one type of representation or inherent properties of the representation, and (c) they facilitate the formation of deeper knowledge structures by enabling the integration of related information from different representations.

While the potentials of MERs to support the learning process have previously been demonstrated, they also place additional demand on the learner, which can increase their cognitive load and even negatively influence the learning process [40]. This is supported by studies pointing towards students’ difficulties with MERs (e.g., [38,41]). Therefore, the use of MERs requires consideration and the careful management of the cognitive load of the learners.

### 2.2. Cognitive Load Theory and Cognitive Theory of Multimedia Learning

When discussing and analyzing learning environments, especially those involving multiple sources of information, theories from psychology regarding the cognitive load of the learner can be used. Here, the use of AR is a prominent example as it can integrate multiple visualizations of information and data into the learners’ existing environment [15,23,42]. Adding extra information into the real environment can thereby be discussed using the cognitive load theory (CLT; [43,44]) and the cognitive theory of multimedia learning (CTML; [45]).

The base assumption of the CLT [43,44] is that of a limited working memory in which the learner must process all provided information in the form of elements and integrate them into their long-term memory, which is assumed to be unlimited. This cognitive load imposed on the learner can thereby be described in three categories: (a) intrinsic cognitive load (ICL) caused by the inherent complexity of the learning content, (b) extraneous cognitive load (ECL) caused by tasks that are not relevant to the learning process, and (c) germane cognitive load (GCL) describing the load created by the learning process itself. To measure these three categories of cognitive load in educational laboratory settings, for example, a questionnaire developed by Thees et al. [46] can be used. Following the assumptions of the CLT, the ECL should be kept minimal to free capacity in the limited working memory for meaningful processing.

The CTML [45] is based on the similar assumption of a limited working memory with an additional emphasis on the learner being required to actively integrate the perceived information into mental representations to achieve meaningful learning. This processing can be described in three parts: selection of relevant information, organization of this information, and integration into the existing knowledge and long-term memory. As, like in the CLT, the capacity of the working memory is assumed to be limited, the CTML also targets a reduction of learning irrelevant cognitive load and proposes multiple design principles to prevent cognitive overload [47].

Regarding the use of AR, the two principles of temporal and spatial contiguity are highly important [47,48]. These suggest that the related information should be presented simultaneously as well as spatially close to each other to promote learning and reduce extraneous processing [49,50]. Similarly, the split-attention effect describes the division of a learner’s attention when information is presented separated from each other. This causes search processes irrelevant to learning to result in an increased ECL [48,51,52,53]. A review by Schroeder and Cenkci [50] confirmed the advantages of integrated design formats compared to separated formats and strengthened the split-attention effect as a rather stable finding in multimedia research.

Following these theories, AR can be an important tool in the optimization of learning environments. An ideal AR environment should therefore visualize related information in a temporally and spatially coherent manner to minimize the learning of irrelevant cognitive load. Here, AR offers particular advantages in areas where such integration would otherwise be impossible or difficult, as is the case in laboratory experiments.

### 2.3. Sensors for Augmented Reality

While AR environments, in contrast to VR environments, are characterized by the integration of virtual visualizations into the real environment, this integration also leads to major problems and requires sensors for seamless integration [25]. It is essential for a good user experience that the integration is robust and accurate, whereby the sensors used for integration can be roughly divided into two groups.

The first group consists of sensors that detect the general environment of the user. These collect basic information needed for an AR environment, such as the position of the device used for AR. In the case of head-mounted displays (HMDs), this typically represents the position of the head in the room, which is used to render accurate AR environments on the devices’ displays. Besides such general environmental recognition, which is independent of the content of the AR environment, individual applications may require additional information for their visualizations. For example, it may be necessary to recognize surfaces to display a visualization on a table or to fixate virtual windows to a wall. However, it is also possible that real objects should be integrated into the AR environment. One example of this is the experimental components we use, which represent anchor points for associated visualizations. Such integrations require an even finer detection of the environment, which not only recognizes it in its entirety but also clearly identifies and localizes individual components.

The second group continues this detailed environment recognition and describes application-specific sensors. Thus, in the context of physical experiments such as those considered here, not only is the detection of the position of the experiment with its individual components required, but also the physical measured values to be observed in the experiment have to be acquired using additional sensors. Such sensors collect the measurement values in the actual experiment and transmit them to the AR device for visualization and integration into the virtual environment. Examples of such measured values are voltages and currents in electrical circuits or temperature measurements in experiments on heat conduction. In addition to physical measurements, other experiment-specific data can also be collected by such sensors. Here, the wiring of the built-up circuit would be an example.

Because of the differences between application-independent and application-specific sensors, there are also significant differences in the availability of the required sensors. Most devices used for AR natively integrate sensors or software solutions to detect the position of the device in space and place virtual content within it. There are also various solutions for localizing real objects in the environment, e.g., by means of visual markers. However, there are typically no sensors available off-the-shelf that collect application-specific data and are optimized for AR applications.

In the context of physics experiments that integrate measurement values into an AR environment, Thees et al. [18] implemented an AR environment for an experiment on heat conduction in metals using a Microsoft HoloLens (first generation). They used the sensors and software provided by the HoloLens to generate the application-independent parts of the AR environment and to enable interactions with it. The real-world experimental setup was recognized using visual markers and the Vuforia software package with the visualizations attached to this marker. The physical measurements in the form of the temperature profile along the investigated metal rod were acquired using a traditional thermal imaging camera. For this purpose, in addition to the smartglasses themselves, another computer was required which controlled the thermal imaging camera; this offered the possibility to configure the measurement apparatus and extract the measured values. These were then transmitted to the smartglasses via WLAN and visualized.

Sonntag et al. [54] also used a Microsoft HoloLens (first generation) for their AR applications for a capacitor, an electron beam deflection tube, and a Teltron tube with Helmholtz coils to create the application-independent parts of the AR environment and to enable interactions with it [55]. However, they identified the position of the experimental setup by means of a visual recognition based on a CAD model, with an option to manually correct the recognized position. The physical values measured in the experiment were recorded by means of a multimeter connected to a Raspberry Pi via USB. The data were then also transmitted via WLAN to the smartglasses for visualization.

Both approaches, however, show significant limitations. The first and most prominent limitation is the fact that they rely on a separate computer combined with traditional measurement sensors to collect the measurement values and forward them to the smartglasses. In addition, both types of position identification have limitations. The position determination which is used by Albuquerque et al. [55] has the advantage that no disturbing visual markers are needed in the experiment, but the mention and implementation of a manual correction of the position indicate low accuracy. In addition, positioning is done only once at the beginning of the experiment and thus cannot react to a change of position during the experiment. The detection of visual markers used by Thees et al. [18] has advantages here as it can achieve higher accuracy and also detect a changed position during experimentation. However, it requires sufficiently large and prominent visual makers on the experimental setup. In addition, the marker must be clearly and unambiguously in the field of view of the tracking camera, which is not guaranteed during the entire experiment. Just as in the case of Albuquerque et al. [55], this limitation is minimized by the fact that in the case of a loss of recognition of the visual marker, its position can be kept stationary by means of the environment recognition of the HoloLens, and the experiment setup itself is mostly static.

Based on these observations, the goal of the development of the sensors presented here was to eliminate these limitations as part of a new experiment. For this purpose, new sensors were developed to measure the physical values of voltage and current in the electrical circuit and transmit the collected measured values to the smartglasses without an additional computer. In addition, a new system for the position recognition of experimental components based on SteamVR was developed (see Section 3.3) which can permanently determine the position of the components during experimentation with high accuracy and precision. Furthermore, a cable identification system was developed to track cable connections between sensor boxes, allowing real-time circuit reconstruction.

### 2.4. SteamVR Locating System

This section provides an overview of the basic functionality, as well as the available software and hardware implementations of the SteamVR positioning system—the technology we use for our positioning system.

In general, SteamVR is a tool developed by Valve that supports the creation of content for virtual reality settings (https://www.steamvr.com/, accessed on 28 October 2021). Besides a collection of software tools, it also includes a hardware solution for the localization of VR headsets (https://partner.steamgames.com/vrlicensing, accessed on 28 October 2021). This system consists of stationary base stations emitting infrared light beams and an array of sensors located on a tracked object, such as the HMD or controller, that receive these beams. Based on the information received by the tracked object, its position can then be deduced.

The first generation of this localization system uses the time difference between two infrared pulses to deduce the position of the sensor in relation to the base station. For this, the base station contains two rotors whose rotational axes are aligned with the x- and y-axes of the base station. The rotors spin at a constant speed, and each projects an infrared light beam into the room. Additionally, the base station contains an array of infrared LEDs that project infrared light into the whole room. To locate a sensor in the room, the base station first flashes a synchronization pulse into the whole room, indicating the start of the rotation of each rotor. After sensing this flash, the sensors time the duration between the initial flash and the time the light-beam projected by the rotors reaches the sensor. Based on this time difference and the constant rotation speed of the rotors, their angles can be calculated. This results in two known angles relative to the base station that describe the direction from the base station to each sensor. Combining multiple sensors in a known arrangement, the 3D position of the tracked object can then be deduced with an optional second base station, increasing accuracy and reducing occlusion.

The second generation of the localization system, which is used in this work, reduces the base station to a single rotor with no LED array. This is achieved by placing two lenses offset by $120^{\circ }$ on one rotor that projects two light beams angled at $\pm 30^{\circ }$ to the rotational axis. Information about the current rotation angle of the rotor is encoded into the emitted infrared light-beam instead of relying on a synchronization flash. Each localization sensor can decode this angle information from the two beams. With both angles known, the direction from the base station to the sensor can be calculated again. The location reconstruction using multiple sensors and multiple base stations follows the same process as that used in the first generation.

The first generation of the positioning system was first used by the HTC Vive VR headset (https://www.htc.com/us/newsroom/2016-06-07/, accessed on 28 October 2021). The second generation was first used by the HTC Vive Pro (https://www.vive.com/us/product/vive-pro-full-kit/, accessed on 28 October 2021) and has since been adopted by multiple other HMDs such as the Valve Index (https://store.steampowered.com/valveindex, accessed on 28 October 2021) and the HTC Vive Pro 2 (https://www.vive.com/us/product/vive-pro2/overview/, accessed on 28 October 2021).

Besides the official implementations of the localization system, multiple open-source implementations of sensors as well as localization frameworks have been developed. One example of this is libsurvive (https://github.com/cntools/libsurvive/, accessed on 28 October 2021), which is a set of tools and libraries and offers an open-source implementation for the localization of SteamVR devices. These use the raw data stream provided by the devices over USB containing the timing information of each sensor to calculate the location of the supported devices, and this approach is compatible with both the first as well as the second generation of SteamVR. Thus, the goal of libsurvive is to replicate the location functionality offered by the official SteamVR software stack.

Besides projects targeting official SteamVR devices, there also exist multiple projects that have developed their custom hardware using the locating system. The Tundra Tracker (https://www.kickstarter.com/projects/tundralabs/tundra-tracker, accessed on 28 October 2021), for example, presents an alternative to the official VIVE Tracker (https://www.vive.com/de/accessory/vive-tracker/, accessed on 28 October 2021) and integrates the SteamVR tracking system into a trackable object, which can be tracked independently from the main SteamVR device. The Tundra Tracker thus claims to be smaller and cheaper while also allowing integration into the official SteamVR software stack for localization. Another similar third-party tracking device is the Manus SteamVR Pro Tracker (https://www.manus-vr.com/steamvr-pro-tracker, accessed on 28 October 2021).

Besides these projects still targeting VR, the original use case of the system, other projects are developing custom hardware as well as software to use the localization system in other areas. Here, bitcraze, for example, offers a positioning system based on the SteamVR base stations to locate their Crazyflie drones (https://www.bitcraze.io/documentation/lighthouse/, accessed on 28 October 2021). The system consists of a custom Lighthouse positioning deck (https://store.bitcraze.io/collections/positioning/products/lighthouse-positioning-deck, accessed on 28 October 2021) with four sensors receiving the infrared light-beams from SteamVR base stations and a custom firmware converting the received light-beams into location data.

The implementation of our positioning system based on SteamVR 2.0 is presented in Section 3.3 (hardware) and Section 4.4 (software).

## 3. Hardware for the Smart Learning Environment

This section describes the design of a total of three different sensors that are integrated into our smart sensor boxes for electrical components to enable our smart learning environment for electrical experiments. The sensors consist of a circuit measuring the voltage across and the current through the electrical component inside the box, the cables used to construct the circuit, and a localization system using SteamVR 2.0.

### 3.1. Electrical Voltage and Current Measurement

The first sensing unit integrated into our sensor box is used to measure the voltage across and the current through the electrical component integrated into the box. This sensing unit has been designed to be agnostic to the type of component to enable flexible usage for various electrical experiments. To fit most electrical experiments dealing with safe-to-touch voltages, the specifications regarding the voltage and current range measured by the unit were been determined to be $V_{IN}=-15\;V\;\ldots \;+15\;V$ and $I_{IN}=-500\;\mathrm{mA}\;\ldots \;+500\;\mathrm{mA}$, respectively.

**Figure 1:**
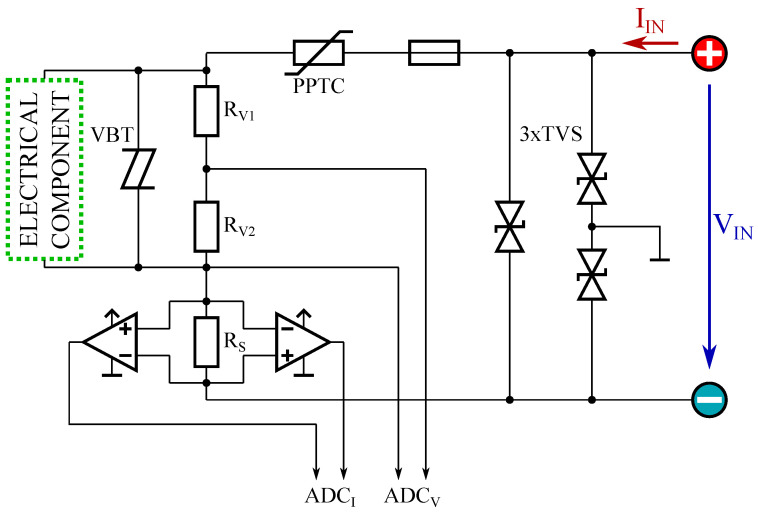
Circuitry of the voltage and current measurement.

Figure 1 shows the circuitry of the measurement unit together with additional components for damage protection. This damage protection was added to ensure reliable operation in every possible use case and to make the sensor boxes robust against improper use and unwanted negative effects. Three transient voltage suppressor diodes (TVS diodes) are included to protect the internal circuitry from external electrostatic discharge (ESD). To protect the circuitry against applied voltages and currents beyond the specified ranges, a voltage-triggered bidirectional thyristor (VBT) and a polymeric positive temperature coefficient (PPTC) have been included.

For voltage measurement, a voltage divider formed by two precision resistors $R_{V1}$ and $R_{V2}$ is used to map the applied voltage range to the differential input range of the analog to digital converter (ADC) $ADC_{V}$. The current measurement is realized by measuring the voltage across the shunt resistor $R_{S}$. This voltage is amplified by two anti-parallel instrumentation amplifiers and routed to the differential inputs of $ADC_{I}$. As instrumentation amplifiers, current shunt monitors have been used which are characterized by a large range of bidirectional common-mode voltage. A sample rate of 4 ms is scheduled for the ADCs; therefore, the analog signals are low pass filtered in the hardware before they reach the ADC inputs. The sample rate can then be reduced in software to provide smoother data output.

### 3.2. Cable Identification

The identification of cables is based on the one-wire protocol (https://www.maximintegrated.com/en/products/ibutton-one-wire.html, accessed on 28 October 2021). Every plug forms a one-wire slave with a unique 64-bit identifier (ID), with each socket representing a one-wire master which reads out the IDs of all the plugs connected to it. With the help of a predefined list stored in software, two plugs are uniquely assigned to one cable. On account of the structure being a bus protocol that supports several slaves per master, “multi-stacking plugs” can also be realized.

Technically, there is a 1 kBit EEPROM chip (DS2431 from Maxim) integrated into each plug, which only requires two connections for both data transmission and power supply. This allows the use of a robust 6.35 mm audio jack with three contacts (two for the cable identifier and one for the experimental circuit). Currently, only the 64-bit identification ID of the one-wire interface on the DS2431 is used to identify the plug.

The master that reads the IDs can be implemented either on a microcontroller or directly on the FPGA, as in our case, depending on the available resources. Reading a single 64-bit ID on the bus takes approximately 15 ms, which means that even with several slaves connected or in multiplex configurations (several sockets share one one-wire master), a very responsive behavior is possible.

### 3.3. Position Identification

Even if there already exists a range of tracking devices utilizing the SteamVR system (see Section 2.4), they mainly target 3D position tracking. Furthermore, they usually integrate an additional inertial measurement unit (IMU) and advanced sensor fusion algorithms to achieve fast updating and accurate positioning information in 3D space. Since our sensor boxes are usually located on a table, moving on a 2D plane, and the requirement for update rates are not quite as high as in VR, we developed our own implementation of a tracking device with a focus on a simple and cost-effective implementation.

To benefit from the improved accuracy and the reduced susceptibility to interference compared to the first generation of the SteamVR tracking system, our system is built around the base stations from the second generation of SteamVR, designated as SteamVR base station 2.0 (BS2). As already described in Section 2.4, there is no publicly available, official documentation of the system, because of which the following information about the BS2 and the protocols used is based on knowledge from open-source projects such as the libsurvive project and the crazyflies Lighthouse positioning deck introduced in Section 2.4 as well as targeted reverse engineering.

#### 3.3.1. Hardware of the SteamVR 2.0 Base Stations

The internal setup of the BS2 consists of two lenses offset by $120^{\circ }$ on a single rotor which are used to project two $\pm 30^{\circ }$ angled planes called light-beams. The infrared light needed for the generation of the light beams is modulated by the biphase mark code (BMC) decoded output of a 17-bit linear-feedback shift register (LFSR) clocked at 6 MHz. The LFSR is configured with well-defined feedback polynomials and resets to the start seed 1 at the beginning of each new revolution of the rotor. The feedback polynomials and, therefore, the resulting output sequence of the LFSR were specifically designed so that the exact position within the LFSR output sequence can be determined if at least 18 consecutive bits are received. By additionally knowing the rotation speed of the rotor, it is thereby possible to determine the current rotor position in degrees relative to the reset position of the LFSR. To enable the use of several base stations at the same time, the base stations can be operated on different channels. Each channel has two different feedback polynomials and a corresponding rotor speed assigned to minimize potential interference. By alternating the two feedback polynomials of one channel, the base station transmits additional information—so-called omnidirectional optical transmitter data (OOTX-Data). The OOTX-Data contain a preamble, a CRC32 checksum, and a payload of 43 bytes with information such as the serial number and factory measured calibration data of the emitting base station.

#### 3.3.2. Lightbeam Receiver

All of our sensor boxes are equipped with six light sensors, each consisting of a light-to-digital converter IC (TS4231—Triad Semiconductor, Winston-Salem, NC, USA) and a photodiode (BPW34—Vishay Semiconductors, Malvern, PA, USA) to receive the infrared signals. The data of the light sensors are collected and pre-processed in a small field-programmable gate array (FPGA; iCE40HX8K—Lattice Semiconductor Corporation, Hillsboro, OR, USA) and then passed on to the main microcontroller (nRF52840—Nordic Semiconductor, Trondheim, Norway) via the Universal Asynchronous Receiver Transmitter (UART) protocol for further processing. All time-critical tasks such as searching for the position on the LFSR sequence and measuring time differences are carried out on the FPGA due to its excellent capabilities for parallel computing. Control flow-heavy tasks such as sorting and filtering are computed on the microcontroller.

#### 3.3.3. Pre-Calculation for Position Detection

Even though the final calculation of the relative position from the sensor box to the base station is conducted in software on the Microsoft Hololens 2, extensive pre-calculation is performed on the embedded system inside the sensor box.

Once the light sensor gets hit by a light beam, the FPGA starts sampling and decoding the received bits. As soon as enough bits are received, the sequence is checked against the different feedback polynomials depending on the selected channel. If there is a match, a LFSR configured with the detected feedback polynomial and the start value 1 is started, counting the number of cycles until the LFSR has the same value as the received sequence. This number of cycles, the detected polynomial, and the ID of the receiving sensor, as well as the timing information about the length of the light beam and the time difference to the previous light-beam, are transferred to the microcontroller. The microcontroller groups the different light-beam events into pairs of two light beams, detected by the same receiver from the same revolution of the base station. A valid packet thus consists of the number of steps on the LFSR until the hit of the first $-30^{\circ }$ rotated plane on the photodiode and the number of steps until the hit of the second $+30^{\circ }$ rotated plane as well as the ID of the photodiode. Since external influences such as reflections or other infrared light sources can cause interference, the light pulses are filtered and checked for plausibility with several rules to be fulfilled. The filtered packets are then sent together with the other sensor data via BLE. In addition, the microcontroller is responsible for collecting and decoding the OOTX-Data. As soon as a complete and verified packet is received, it is also forwarded via BLE.

### 3.4. Data Communication

Data communication between the Microsoft HoloLens 2 and the sensor box is realized via Bluetooth Low Energy (BLE). For this purpose, a nRF52840 System On Chip (SoC) from Nordic Semiconductor (https://www.nordicsemi.com/Products/nRF52840, accessed on 28 October 2021) is integrated into the sensor box. Besides the actual sending and receiving of the BLE packets, it also takes over the pre-processing and packaging of the acquired sensor data. For each category of sensor data (current/voltage measurement values, cable identification, and position), there are predefined packet structures with special headers to simplify further processing. Furthermore, a battery status indicator, the control of the status LED, and a software update functionality are implemented through the corresponding BLE services.

## 4. Data Processing

In this section, the procedure of processing the sensor data acquired by our custom sensor boxes and presenting them in augmented reality using the Unity game development engine (https://unity.com/, accessed on 28 October 2021) running on a Microsoft HoloLens 2 (https://www.microsoft.com/en-us/hololens/, accessed on 28 October 2021) is described. While only an application for the Microsoft HoloLens 2 is presented here, the communication library and all further processing functionalities are also compatible with other platforms.

### 4.1. Data Communication

As Unity does not provide native support for Bluetooth Low Energy (BLE) used for data communication, a custom plugin was developed. It provides a cross-platform interface to connect to sensors, enabling data communication and processing for the rest of the application.

Different libraries and APIs are used to implement BLE communication, depending on the target platform. For the Universal Windows Platform (UWP) used for development on the Microsoft HoloLens 2, the Bluetooth Low Energy APIs provided by the UWP environment are used. On Windows, a modified version of the *BleWinrtDll* Unity Plugin by Adam Brunnmeier (https://github.com/adabru/BleWinrtDll, accessed on 28 October 2021) is used which utilizes the Win32 Bluetooth API. For support on Android and iOS the *Bluetooth LE Unity Plugin* by Shatalmic, llc is utilized (https://assetstore.unity.com/packages/tools/network/bluetooth-le-for-ios-tvos-and-android-26661, accessed on 28 October 2021). Based on the current build target, the correct hardware layer is automatically chosen, resulting in a uniform development environment for the rest of the application.

### 4.2. Electrical Voltage and Current Measurement

The voltage and current measurement data provided by the sensors are wrapped in a custom data format to achieve high data throughput, requiring additional processing. The incoming bytes are separated into 8-bit timestamps of the newest measurements taken on the sensor and six blocks of 24 bits each representing one measurement. The received timestamp of the sensor is first converted into a local timestamp based on the difference between the new timestamp and the timestamp of the previous package. If no previous timestamp was received, we define the incoming timestamp as corresponding with the current time on the receiving device. After deriving the local timestamp of the newest measurement, the measurement data blocks are processed. For this, the 24 bits of each block are separated into 12 bits for each voltage and current measurement and then converted into the actual measurement values using a predefined offset and correction of the decimal place. The timestamp of this measurement is derived from the calculated timestamp of the newest measurement value and the defined data rate of 24 ms.

Following this process, the measurement data can be used in the rest of the application. The application used in our evaluation study (see Section 5.2) visualizes the current measurement data attached to each component in the circuit in two ways (decimal value and needle deflection). The measurements are also used during circuit reconstruction.

### 4.3. Circuit Reconstruction

To reconstruct an electric circuit assembled by a user, we use the voltage and current measurements combined with the cable identification data provided by the sensors. From this information, we derive the electric potential and current throughout the circuit as well as a representation of the components’ mutual interconnections. We assume a circuit with a single power supply (or battery) and cables with negligible internal resistance; i.e., the voltage across any cable is zero. We also define the potential of the negative terminal of the power supply as zero and as increasing towards the positive terminal.

Based on the assumption that interconnected cables have the same potential, the general idea of our circuit reconstruction algorithm is to first find groups of cables that are connected to each other and then to calculate the potential of that group by compiling a list of sensor boxes that lie between that group and the negative terminal of the battery.

To identify groups of identical potential, we iterate through all cables with at least one connected plug and check if the cable is connected to an already known cable group. Cables are connected if they share a terminal in one of the sensor boxes. If so, we add the cable to that cable group. If not, we create a new group with unknown potential. If both plugs are connected to different potential groups, we merge them. Evaluating all cables in the circuit results in a list of cable groups with every cable assigned to exactly one group.

To find the physical potential of these groups, we use a breadth-first search starting at the group that is connected to the negative terminal of the voltage source. For each layer of cable group found, we note the sensor boxes leading to it from the negative terminal of the power supply. With our assumption that the potential of the negative terminal of the power supply is zero, the potential can be calculated as the sum of voltages from the sensor boxes leading to it. As the sensors measure positive and negative voltage and current, they define a positive and a negative terminal. While calculating the potential of the group, we, therefore, have to check the polarity in which the box is integrated into the circuit. Furthermore, the breadth-first search may reach a box from the side that is further away from the negative terminal of the power supply. However, in real circuits, and if the power supply voltage is positive, the potential can only increase across a sensor box. Likewise, it has to decrease if the power supply voltage is negative. Therefore, we can identify the polarity of a sensor box and whether the search reached a box from the wrong side by checking the polarity of the terminal adjacent to the currently evaluated potential group. The resulting set of cable groups and their interconnections starting at the negative terminal of the voltage source are characteristic of a particular circuit and can be used for further processing.

Following the potential identification across the circuit, we also identify the current through each cable. For this, we currently assume each cable to have at least one end that is connected exclusively to one terminal of a sensor box with no other cables being connected to the same terminal. Thus, the current through the cable must be identical to the current through the sensor box connected to that end. If only one end of the cable is connected to the circuit, the current through the cable must be zero. Physically, the direction of the cable is irrelevant. However, in software, the plugs of the cables have unique IDs, so we need to check the direction of the cable to assign the correct sign to the electric current. In a real experiment, each cable group is connected to terminals with an electron current into the group that we define as inlets and to other terminals with an electron current out of the group (outlets). In software, inlets and outlets can be distinguished by the order of the breadth-first search because the negative terminal of the battery is always the only inlet for the first cable group. Combined with the information of which plug is connected to which sensor box, we can assign a sensor box to both ends of any cable and the value and sign of the electric current flowing through it. While the algorithm internally uses the physical direction of electric current for calculation, the technical current direction can easily be deduced by inverting the signs.

### 4.4. Position Identification

As each incoming data package containing position information from the sensor box can contain the information of multiple light sensors, it is first separated into multiple blocks, each containing one sensor. Following this separation, all blocks are processed in sequence, and the contained timing information (i.e. the number of steps on the LFSR for each plane) is saved as the last measurement value for this sensor. In addition to the new time values, we flag whether every existing sensor was updated in this data package.

At every frame rendered by the Unity application, every currently tracked sensor box is evaluated, and if new values are received, the location of the sensor box is updated.

#### 4.4.1. Direction Calculation

The first step in locating the sensor box is the calculation of the direction from the stationary base station to each of the six light sensors on the box currently receiving light beams.

The base of our calculations is the received number of steps on the LFSR describing the rotor position at the two planes projected by the base station at each light sensor, as received over BLE. While the rotation speed varies with the current channel of the base station, this speed is constant and known for all possible channels. The received steps can therefore be easily converted into the rotation angle of the projected line in relation to the starting position at $0^{\circ }$. We thereby define α as the angle of the first plane rotated $-30^{\circ }$ off-axis and β as the angle of the second plane rotated $+30^{\circ }$ off-axis with the rotors’ period of the current channel.

(1) $$\alpha =\frac{\pi }{\mathrm{period}}-\frac{2}{3}\pi$$

(2) $$\beta =\frac{\pi }{\mathrm{period}}-\frac{4}{3}\pi$$

As the distance of the sensor to the base station is unknown, the two light beams received by the sensor form planes on which the sensor must be located. We can define the normals of these possible planes in the coordinate system of the base station as follows, with $n_{1}$ being the plane created by the first light beam and $n_{2}$ being the plane of the second light beam.

(3) $$\vec{n_{1}}=\left(\begin{array}{c} cos(\alpha )\\ tan(\pi /6)\\ sin(\alpha ) \end{array}\right)$$

(4) $$\vec{n_{2}}=\left(\begin{array}{c} -cos(\beta )\\ tan(\pi /6)\\ -sin(\beta ) \end{array}\right)$$

With the sensor needing to be located on both planes, their intersection results in a line describing the direction to the sensor in the local coordinate system of the base station. This direction can be calculated directly using the cross-product of the plane normals.

(5) $$\vec{dir}=\vec{n_{1}}\times \vec{n_{2}}$$

Due to the physical setup of the base station (e.g., refraction of the projected light-beams at the front window of the base station) as well as manufacturing tolerances, with the sensor direction calculated based on the raw timing, the data contain significant inaccuracies. To increase the accuracy, each base station is calibrated during manufacturing with the resulting calibration parameters being written to the permanent storage on the base station. These values include, for example, corrections for the position of the lenses on the rotor and the tilt of each plane as well as distortion parameters. They are sent from the base station to the sensors using a separate OOTX package as described in Section 3.3. However, these calibration parameters do not describe a correction of the uncalibrated direction, as calculated above, but instead describe the expected rotation angles of both light beams at a known sensor position in relation to the base station.

No details regarding the calibration parameters and their meaning as well as the official correction calculation are publicly shared by Valve. However, the *libsurvive* toolkit integrates a reverse-engineered approach to use the calibration parameters provided by the device to calculate the expected rotation angles of the light-beams (https://github.com/cntools/libsurvive/blob/master/src/survive_reproject_gen2.c, accessed on 28 October 2021). We adopt their general approach, as contained in the source of the toolkit, while adapting it to fit our environment.

To integrate the calibration model into our application, we first use the uncalibrated direction vector as the sensor position to calculate the expected rotation angles $\alpha_{\mathrm{calib}}$ and $\beta_{\mathrm{calib}}$ at this position utilizing the calibration parameters. We then calculate the corrected rotation angles $\alpha_{\mathrm{cor}}$ and $\beta_{\mathrm{cor}}$ by combining the observed as well as the expected rotation angles to a corrected angle incorporating the calibration parameters.

(6) $$\alpha_{\mathrm{cor}}=\alpha +\left(\alpha -\alpha_{\mathrm{calib}}\right)$$

(7) $$\beta_{\mathrm{cor}}=\beta +\left(\beta -\beta_{\mathrm{calib}}\right)$$

Using these corrected rotation angles of the light beams, we are then able to calculate the corrected direction using the plane calculations above. By using the uncalibrated direction as the expected sensor position, we do not follow the intended calibration process and add errors in our correction. However, these errors are significantly smaller than the errors corrected by the approach and proved to be negligible in our use case.

#### 4.4.2. Base Station Position Localization

Using the direction information derived from the infrared sensors (see Section 4.4.1), only the direction in the local reference frame of the base station is known. Therefore, locating the sensor box in real-world coordinates requires the location of the base station to transform the local direction into the real-world reference frame.

While the global position of the base station could be located directly—e.g., using a visual marker attached to the base station—this does not seem practical for every use case. To achieve ideal tracking, the base station should be located above the tracked area while pointing at it. This means that the base station is not directly accessible and, for example, a visual marker is difficult to place and recognize. In addition, the localization accuracy of the visual marker might not be good enough and may be reduced due to the distance to the base station.

Therefore, we implemented an indirect localization of the base station for initialization. For this, the base station is placed at any point in the room while covering the area we want the localization to work in. During initialization, we now use one sensor box as the reference sensor with a known position in the global reference frame. This position can be identified in multiple ways; in the application used in our evaluation study (see Section 5.2), we used a QR code temporarily placed on top of the sensor box, which is located using the built-in QR code localization of the Microsoft HoloLens 2 (https://docs.microsoft.com/en-us/windows/mixed-reality/develop/platform-capabilities-and-apis/qr-code-tracking, accessed on 8 October 2021). We then locate the base station in the local coordinate system of the reference sensor and derive the global location of the base station using the known global location of the reference sensor.

To locate the base station in the local coordinate system of the reference sensor, we first make an initial guess regarding its position. Our first guess regarding the rotation of the base station is derived from the accelerometer built into the base station, which is transmitted to the sensors together with the calibration data in the OOTX package (see Section 3.3). Based on these readings, we can derive the orientation of the base station to the ground in the global reference frame. The next step uses the calculated direction to each light sensor in the reference frame of the base station combined with the first rotation guess by projecting the directions onto a plane parallel to the ground and calculating the angle of each projected direction with regards to the forward direction of the base station. After this transformation, we can sort the sensors by angle and derive the order in which they are positioned in relation to the base station. As the order in which the six light sensors on the sensor box are hit differs depending on the rotation of the sensor in relation to the base station, we can derive in which direction from the reference sensor the base station is placed. This enables us to obtain an initial guess regarding the position of the base station. As our use case consists of tracking on a table, we guess the distance to the base station to be 0.5 m away from the sensor box and 1 m above it. We also assume the base station to be directly facing the reference sensor box and therefore rotate the initial rotation guess of the base station accordingly.

After this rough initial guess of the location of the base station, we use OpenCV (https://opencv.org/, accessed on 28 October 2021) for optimization. Thus, we assume the base station to be an ideal pinhole camera with no distortion. This allows us to calculate the location of each light sensor on a virtual image created by the ideal camera using the known direction from the base station. With these image points, the intrinsics of our ideal pinhole camera, and the known arrangement of the sensors on the real sensor box, we can then use the iterative solvePNP function (https://docs.opencv.org/3.4/d9/d0c/group__calib3d.html#ga549c2075fac14829ff4a58bc931c033d, accessed on 28 October 2021) provided by OpenCV to optimize the position and pose of the base station in the local reference frame of the reference sensor box. This location can then be transformed into a global location using the known global location of the reference sensor.

As this calculation of the position of the base station is sensitive to noise, we collect multiple positions during initialization. The final location of the base station is then defined as the average location of the collected positions after removing outliers that might occur due to issues with the initial guess, measurement errors, or optimization errors by OpenCV.

#### 4.4.3. Positioning on Table

After initialization and locating the base station in the global reference frame, we can use the calculated sensor directions of each sensor box to locate the sensor box on a table. For this, we additionally initialize a plane representing the table while initializing the location of the base station. This is simply achieved by placing the reference sensor used during base station localization on the experiment table. As the global coordinate system is always aligned horizontally when using the Microsoft HoloLens 2, we only require the height of the table to define the table plane.

Due to the horizontal alignment of the global coordinate system, locating a sensor on the table plane is then reduced to calculating the 3D position of the direction vector from the base station to the sensor at the height of the table after transforming the initially local direction to the sensor into the global reference frame.

Following the location of all sensors on the table plane on each new frame of the application, the position and rotation of the sensor box can simply be derived from the known sensor arrangement. If only one sensor provided position data, only the position of the box can be derived, with the rotation remaining unknown and left unchanged from the previous location. As soon as two sensors provide position data, the rotation of the box can also be derived. If more than the necessary number of sensors provide new data, the position and rotation of the sensor box are set to the mean of all position and rotation data derived. To reduce jumps due to errors in tracking and to smooth the position of the sensor box, additional filtering such as the detection of unexpected jumps in position is applied before updating the sensor box position in the final application.

## 5. Evaluation

To assess the newly developed sensors in practice, two types of evaluations were conducted. The first evaluation consisted of multiple setups testing the accuracy and precision of the implemented sensors themselves under laboratory settings. The second evaluation consisted of a field study with students evaluating the usability of the entire system consisting of multiple sensor boxes and including a first AR application running on the Microsoft HoloLens 2.

### 5.1. Sensor Accuracy

Since the accuracy of the data obtained by the various sensors has a significant impact on the usability of the overall system, especially in regard to the STEM experiments targeted, a precise evaluation of the sensors is necessary.

#### 5.1.1. Electrical Voltage and Current Measurement

To determine the accuracy of the current and voltage measurement unit, a set of five identical sensor boxes was considered. To explore the full range of the electrical specification, resistor values of 25 Ω, 50 Ω, and 150 Ω were employed as incorporated electrical components. As a reference, two calibrated precision multi-meter instruments were used for voltage and current measurement, respectively. Measurement data were obtained by tuning the power supply to the desired expected value, validated by the reference multimeters.

**Figure 2:**
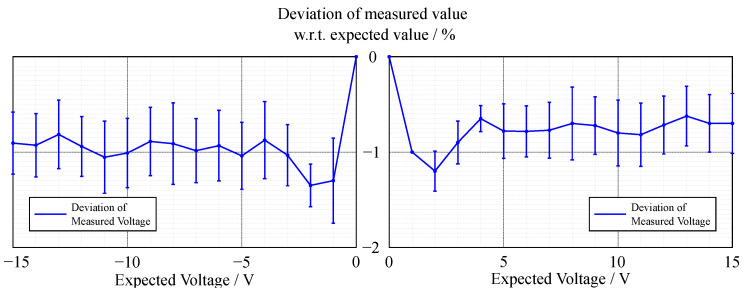
Relative deviation of the voltage as measured by the sensor box with regard to the expected value.

**Figure 3:**
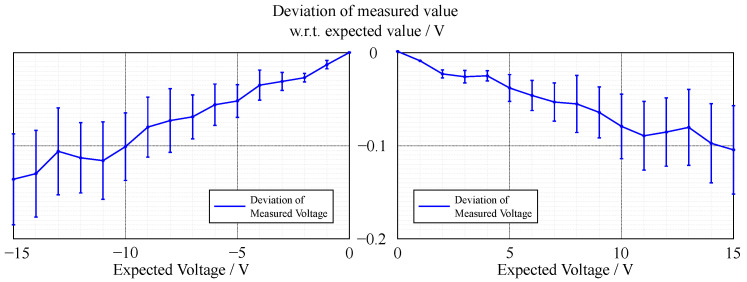
Absolute deviation of the voltage as measured by the sensor box with regard to the expected value.

**Figure 4:**
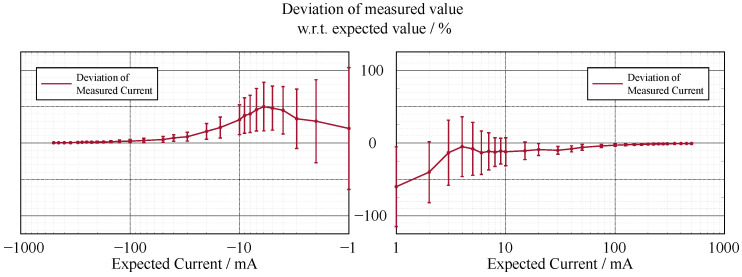
Relative deviation of the current as measured by the sensor box with regard to the expected value.

**Figure 5:**
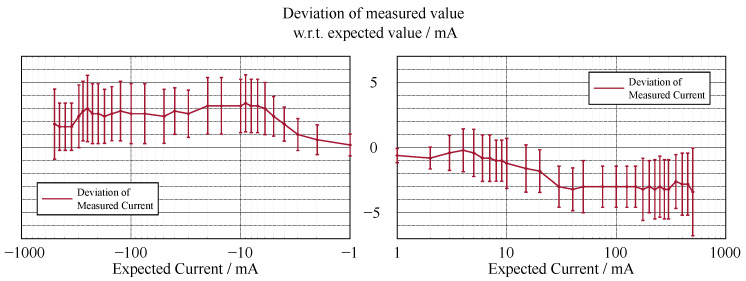
Absolute deviation of the current as measured by the sensor box with regard to the expected value.

In contrast to the accuracy of the voltage measurement (see Figure 2 and Figure 3), the current measurement exhibits a relatively high error in the lower measurement range (see Figure 4 and Figure 5).

#### 5.1.2. Cable Detection

The plugs and the cables are detected with the help of the device ID of the one-wire interface. In addition to the ID, a cyclic-redundancy-check (CRC) is also transmitted so that transmission errors can be detected reliably. In our current implementation, we allow for up to five stacked plugs, resulting in a maximum transmission time of approximately 75 ms to receive all five IDs.

#### 5.1.3. Position

**Figure 6:**
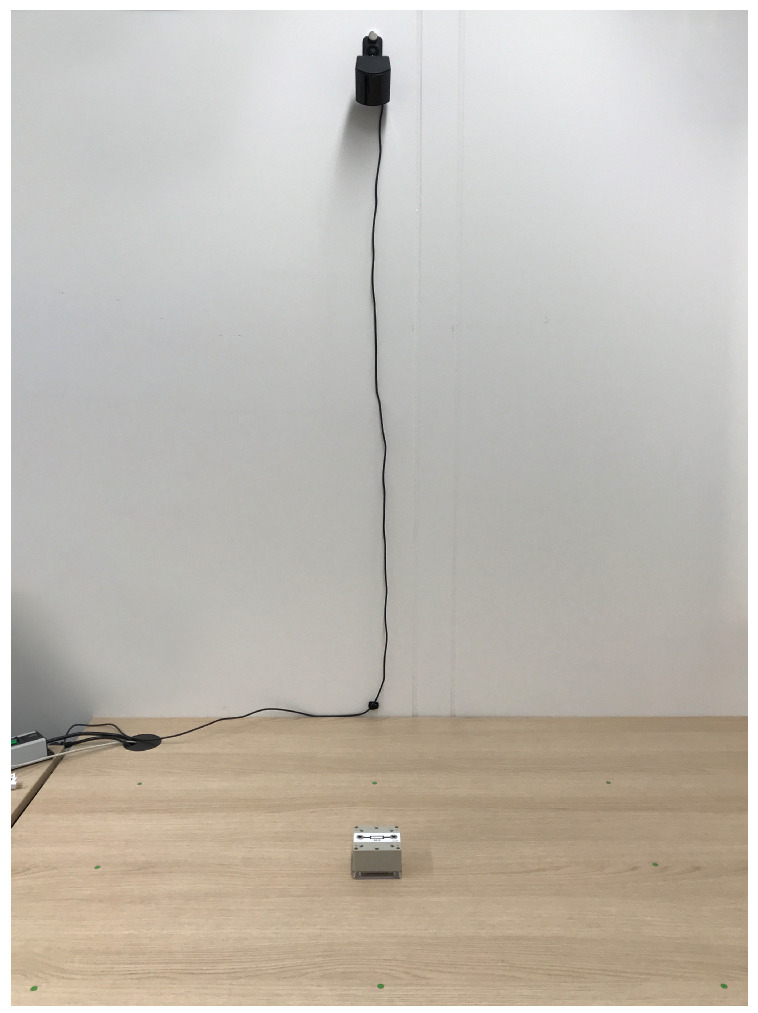
Photo of the experiment setup evaluating the precision of the sensor position on a table.

To evaluate the precision of our positioning system, we placed a sensor box stationary on a table and analyzed the generated position data. The box was positioned at nine points on the table forming a 100 cm × 60 cm grid. This size roughly equates to a typical experiment area. The base station was positioned 50 cm behind and 150 cm above the central position on the grid. A photo of the experimental setup can be found in Figure 6.

After the positioning system was initialized on the central point on the grid, the calculated position and rotation of the sensor box were recorded for 1000 position measurements on each point. Additionally, the raw sensor data consisting of the position on the LFSR sequence were recorded for each of the six light sensors positioned on the sensor box. The identified standard deviation of the position of the sensor box at each point in the grid can be found in Table 1. The standard deviations of the raw LSFR position as means of the six sensors for each box are shown in Table 2.

### 5.2. Usability Evaluation

To evaluate the usability of our newly developed sensors, an initial field study with students conducting typical experiments on basic electrical circuits was conducted. The experimental tasks used in this study are based on previous studies utilizing a previous generation of the sensor boxes presented in this paper [56,57,58,59].

These studies explored the learning gain and cognitive load while conducting experiments exploring basic electrical circuits. The previous generation of sensor boxes used in these studies only contained the circuit for the voltage and current measurements presented here (see Section 3.1). To achieve the localization of the sensor boxes, visual markers were utilized. While these visual markers did not require additional hardware such as a base station to locate the sensors, the tracking quality was low, and the recognition was limited to a distance from the marker of approximately 80 cm. This required the experimenter to purposefully move the tracking camera—i.e. the integrated webcam of the smartglasses—close to the visual marker to achieve tracking. In addition, no cable detection was implemented, with the evaluation of the correct setup of the circuit being conducted by an external expert.

Thees et al. [58] used these sensor boxes to evaluate the learning gain as well as cognitive load in a laboratory study consisting of n=107 German university students whose fields of study were associated with mechanical or biochemical engineering. The participants were randomly assigned to either an AR visualization of the measurement data or visualization of the data on a separate display. The AR visualization utilized the Microsoft HoloLens (first generation) together with the measurement sensors and visual markers to visualize the real-time voltage and current measurement of each component in the circuit integrated with the real experiment. The visualization of the measurement data on a separate display was achieved using an Apple iPad, displaying the measurement values in a well-arranged matrix. Both groups were given the same experimental task consisting of the construction and evaluation of three serial and three parallel circuits, resulting in a total of six circuits. In order to make the testing of the newly developed system as comparable as possible, we also used the same tasks in our study.

The usability of the two visualization systems during these tasks was evaluated using the system usability scale [60], which was filled out by the participants after the experiment. In the analysis, a system usability score of 75.4 (sd 13.6, n=49) was identified for the AR setting utilizing the Microsoft HoloLens (first generation). The separate display setting utilizing an Apple iPad reached a score of 87.7 (s.d. 10.9, n=58) [58]. According to Bangor et al. [61], these values can respectively be described as “good” or “excellent”. Comparing the two ratings using an independent samples t-test revealed a significant difference between the two conditions [58].

In their review of the impact of AR on cognitive load and performance, Buchner et al. [62] noted that especially research targeting see-through AR can be heavily influenced by technical limitations and unfamiliarity with the used system, while, e.g., the use of tablets is mostly familiar. These limitations could result in a high cognitive load due to the technology alone, instead of identifying the effects of an AR presentation. This significant influence of usability is also discussed in previous reviews regarding the use of AR [22,63]. As the study presented by Thees et al. [58] showed differences in system usability between the two groups, their results regarding learning gain as well as cognitive load could also be influenced by the technology. The goal of the study presented here is to explore the usability of the new generation of sensors combined with the Microsoft HoloLens 2 to evaluate its usability in relation to the previous implementations. High usability could thereby reduce the technological limitations of further studies and enable and substantiate further research regarding cognitive load and learning gain when using AR.

#### 5.2.1. Hypotheses and Research Questions

In the evaluation of a previous generation of the sensors, Thees et al. [58] identified lower usability for an integrated AR setting utilizing the Microsoft HoloLens (first generation) compared to a visualization of the measurement data on a separate display. While discussing the results of their study, the small field of view of the smart glasses was noted as one limitation regarding the AR setting. This could also have had an effect on the perceived usability of the system. Additionally, the use of visual markers to locate the component boxes in the environment provided a limitation in the usability of the setup as the location required the learner to purposefully move the tracking camera close to the visual marker to achieve tracking. This limitation did not apply to the separate display setting, as no location of the component boxes was necessary there.

Our new sensor generation combined with the Microsoft HoloLens 2 addresses both of these limitations. Using the new method of position identification using the Steam VR localization system removes inconvenient and error-prone visual marker detection during the experiment, making the localization effortless for the learner. Our new setup also benefits from a significant increase in the field of view of the Microsoft HoloLens 2 (double the area (https://mashable.com/article/microsoft-hololens-2-mwc-2019, accessed on 28 October 2021)) compared to the first-generation Microsoft HoloLens. While not noted as a limitation in the original study, the full hand-tracking on the HoloLens 2 also enabled new modes of interaction, resulting in the easier handling of the device and the hand menu present in our application.

These benefits of the new sensor generation led us to our hypothesis: *The presented sensors combined with a Microsoft HoloLens 2 achieve higher usability compared to the previous generation of sensors combined with the first generation of the Microsoft HoloLens.*

Besides the system usability as a whole, the presence of the Microsoft HoloLens 2 alone might also interfere with the learning process during the experiment. This could also result in a limitation when researching cognitive load and learning gain of AR environments in general. This leads to our research question: *How do the learners rate the interference of the smartglasses during experimentation?*

#### 5.2.2. Participants

In total, 20 participants (17 male, 2 female, 1 diverse/non-binary) took part in the study. All participants were attending the same high school in Germany and had a mean age of 18.2 years (s.d. 2.08 years). They were recruited by contacting all students of the school who took classes in electrical engineering in the upper secondary level (in German: Gymnasiale Oberstufe). Eight students were currently attending the 11th grade, 10 students were attending 12th grade and 2 students were attending the 13th grade. All students had previously been taught the concepts covered in this study as part of their electrical engineering course. The average grade of the students in electrical engineering was 9.9 points (range 0–15 from worst to best, s.d. 4.5), as provided voluntarily, with one participant not providing their grade.

#### 5.2.3. System Setup

**Figure 7:**
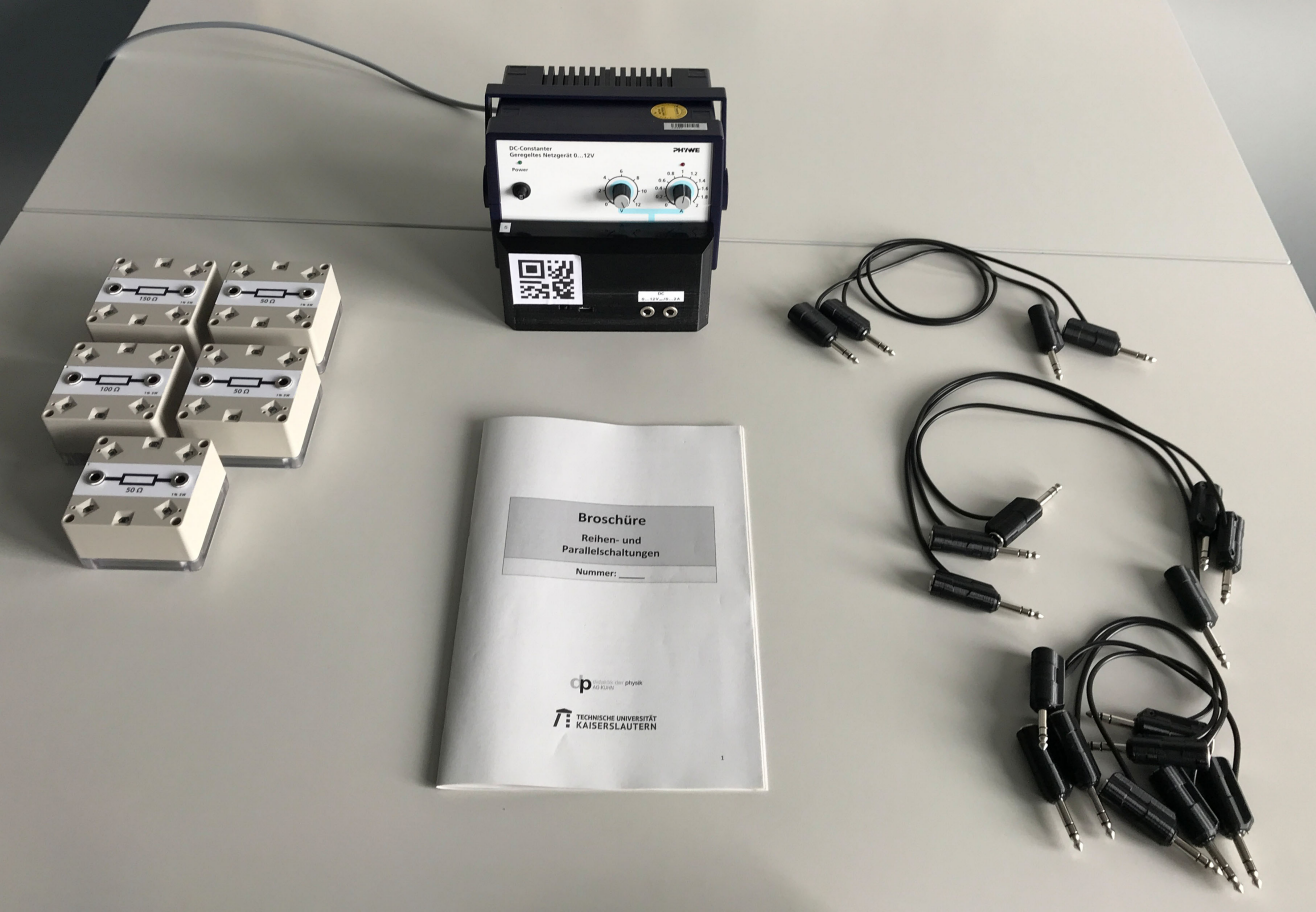
Photo of the experiment setup of the usability evaluation.

The evaluated system consisted of a set of five sensor boxes containing resistors of different values as integrated electrical components as well as a power supply fitted with an identical sensor. To construct the circuits, a total of nine custom cables compatible with the cable detection system were provided (four short cables, three medium cables, and two long cables). As a display device, the Microsoft HoloLens 2 with a custom application was used. A photo of the the experiment setup can be found in Figure 7.

**Figure 8:**
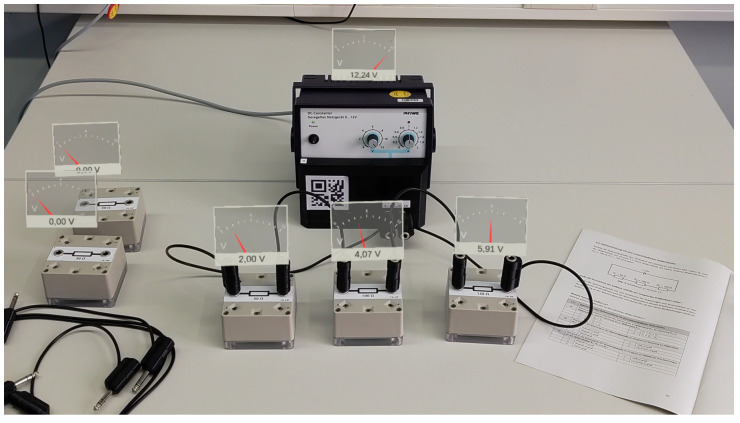
Screenshot of the custom Microsoft HoloLens 2 application captured by the device.

**Figure 9:**
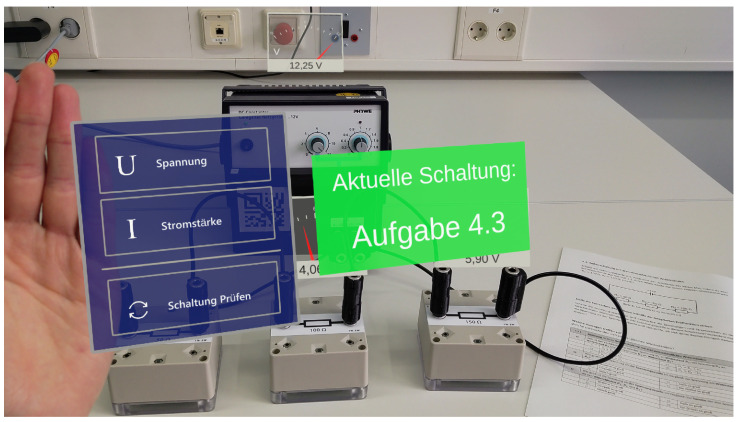
Screenshot of the menu attached to the hand of the participant. The visible options are *Spannung* (voltage), *Stromstärke* (current), and *Schaltung prüfen* (check circuit). As the participant has just checked the circuit, the green window indicates a valid circuit and displays *Aufgabe 4.3* (task 4.3) to confirm the task matching the current circuit setup.

The accompanying Microsoft HoloLens 2 application was developed using the Unity game development engine and received and processed the sensor data. The measurement data were visualized in real-time and fixed to the position of the corresponding sensor box. A screenshot of the visualization can be found in Figure 8. Attached to the hand of the learner was a menu that enabled the learner to switch between the visualization of voltage (in German: *Spannung*) and current (in German: *Stromstärke*). The cable identification implemented by the sensors was utilized to enable an evaluation of the currently constructed circuit. The learner could request the current circuit status using an additional button in the hand menu (in German: *Schaltung überprüfen*). After requesting the circuit status, the learner was either informed that the circuit did not match a given task (in German: *abweichend*) or the matching task number was displayed. A screenshot of the menu and feedback regarding the circuit can be found in Figure 9.

While only one experiment setup with six tasks was evaluated in this study, the application is designed to support a multitude of different experimental setups and tasks. All sensor connections, as well as valid circuits with their display name (e.g., corresponding tasks) and visualizations, are configured using a configuration file. By changing this configuration, multiple different settings can be supported without changing the application itself.

#### 5.2.4. Instruments

To evaluate the usability of the system, the system usability scale (SUS) by Brooke [60] was used as a post-test after the participants completed all the tasks. The participants rated their level of agreement with 10 statements concerning the handling and usefulness of the system on a 5-point scale. The statements were translated to German, and the term “system” was specified as the synergy of the usage of the Microsoft HoloLens 2, the custom software on the device, and the physical experiment setup including the custom sensor boxes. To achieve the final score from 0 (worst) to 100 (best), the individual item scores (value range: 0–4; some items inverted) were added up and multiplied with the factor 2.5, as described by Brooke [60].

Additional to the SUS, three items were developed by Kapp et al. [57] were used to assess the interference caused by the HoloLens 2 during the experiment. The items were used in their original German language and adapted to explicitly target the Microsoft HoloLens 2 instead of the general term “digital assistance system” used by Kapp et al. [57] The students ranked their agreement with the three presented statements on a six-point Likert scale ranging from “does not apply at all” (1) to “fully applies” (6). The final score consists of the combined mean answer value of the items scaled to a value range of [0, 1]. The items of the adapted scale are presented in Table 3.

**Figure 10:**
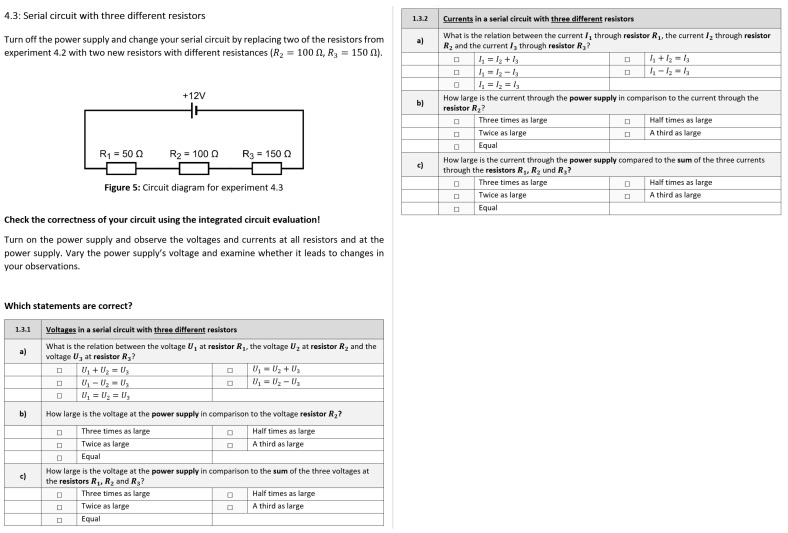
Example task from the study examining a serial circuit consisting of three different resistors (translated for this publication).

The experimental tasks in our study were identical to the study conducted by Thees et al. [58]. Following general information about the experiments, a total of six tasks were presented. Each task consisted of a circuit diagram which had to be constructed by the learner followed by six single-choice questions dealing with the voltage and current at the components of the circuit. Two tasks additionally required the learner to note the measurement values at each component for two different power supply voltages. Three of the tasks consisted of serial circuits, and three tasks were parallel circuits, each with increasing complexity. After the six tasks, a short results section securing the observed relations in each circuit type was to be answered. An example task can be found in Figure 10.

#### 5.2.5. Procedure

After a short introduction to the goals of the study, the participants started by getting an introduction regarding the components used in the study and reading general information about the experiments. After all the questions regarding the experiment setup were answered, the Microsoft HoloLens 2 was introduced, the participants fitted the glasses to their head, and a short introduction into its usage as well as the functionality of the custom application was given by the instructor. As the glasses calibrated themselves to each participant without interaction using the built-in eye-tracking capabilities (https://docs.microsoft.com/en-us/hololens/hololens-release-notes#auto-eye-position-support, accessed on 28 October 2021), no separate calibration was necessary. After familiarization with the device, the participants initialized the position of the sensor boxes. The power supply position was kept stationary during the experiment and was located using a QR code. Following this initialization, the participant started the six experimental tasks with no further assistance. After completing all tasks, the participants removed the glasses and answered the post-test without delay. At the end of the procedure, the demographic data of each participant were collected.

#### 5.2.6. Results

The internal consistency of the system usability scale was $\alpha_{c}=0.43$ (Cronbach’s alpha [64]). The average usability score following the scoring by Brooke [60] was 94.13 with a standard deviation of 4.75. Following the review by Bangor et al. [61], this can be classified as “best imaginable”—the highest possible rating.

The scale used to evaluate the interference by the Microsoft HoloLens 2 showed an internal consistency of $\alpha_{c}=0.52$ (Cronbach’s alpha [64]) with an average score of 0.11 (sd 0.11). The correlation between the interference and the system usability was not significant (r=−0.31, p=0.18).

## 6. Discussion

In this work, we presented a new sensor system for STEM experiments covering basic electrical circuits in educational settings. The sensor system mainly consists of boxes for physical experiments which contain both the electrical component used in the experiment and a custom sensor integrating voltage and current measurement as well as the detection of connected cables and the position of the box. These custom sensors are accompanied by a custom application processing the acquired sensor data and generating corresponding visualizations. A previous generation of these sensor boxes was already used in multiple studies, and their usability was evaluated [56,57,58,59].

### 6.1. Current State and Limitations

The accuracy of the voltage sensing unit proved to be around 1%, with the current measurement having a varying deviation of the measured value in terms of percentage; however, the absolute deviation was around 3 mA. The lower accuracy of the current measurement could be due to two factors. First, the resolution of the current measurement is only 1 mA, which is already 0.2% of the whole measurement range, whereas it is 1 mV for the voltage measurement, which is only 0.007% of the measurement range. Thus, a measurement error that differs in only one least significant bit (LSB) in the measurement results in a larger error. Second, the current shunt monitors have a high measurement error in the range of their zero-crossing, which is similar for likely components that have a comparably high range of bidirectional common-mode voltage. This can be increased by incorporating a larger value for the shunt resistor, which in return limits the measurement range. As the targeted experiments lead to currents in the upper range of the specification, this loss in accuracy in the lower range did not affect the quality of the experiments. Overall, the accuracy and precision of the sensing unit proved to be sufficient.

For the implementation of cable detection, custom cables using audio jacks as plugs were needed. This is a limitation as it prevents the use of standard laboratory cables with banana plugs as connectors when working with our sensor boxes. As the audio jacks used in our custom cables have three contacts, they additionally differ from standard laboratory cables with only one contact providing the electrical connection between boxes. This could cause students to have issues in understanding and using the cables. In addition, the current setup requiring the sensor boxes to identify the cables does not allow for a direct connection between cables without them being plugged into a sensor box. However, the current cable design still allows the stacking of plugs to make parallel connections as it is typically used in laboratory settings. In addition, due to the use of widely available audio jacks as plugs, the components for the custom cables can be acquired cheaply.

Our system for the position identification of the sensor boxes achieved a high precision in our evaluation, with a standard deviation of the position in the x and z-directions of around 0.1 mm and a standard deviation of the rotation of approximately $0.1^{\circ }$. While we were unable to measure the absolute accuracy of the positioning system, a visual inspection of the accuracy when using a Microsoft HoloLens 2 showed similar accuracy to the recognition of a visual marker and proved to be sufficient for our settings. The total system accuracy also might currently be limited by the accuracy of the initialization process currently conducted utilizing a visual marker. For an in-depth analysis of tracking accuracy, a more sophisticated reference system is required [65]. Besides the sensor boxes themselves, our position identification system also requires an additional, stationary base station. This is a significant limitation compared to the use of visual markers, which only require the camera built into the visualizing device to recognize their position. Additionally, our system is currently limited to tracking the position on a 2D plane—e.g., a table—while visual marker recognition typically enables full 3D tracking of the marker. However, this limitation is mostly conditioned by the setup of the experiments used, which are typically conducted on a table. The general approach of calculating the sensor position can also be extended to 3D tracking, as demonstrated by the use of the system in VR headsets and drones (https://www.bitcraze.io/documentation/lighthouse/, accessed on 28 October 2021). An advantage of our system is the increased update rate of the position of the sensor boxes as well as the lack of dependence on visual recognition of the markers. The visual tracking of markers has the limitation that it only works if the marker is large enough and in the field of view of the camera. Especially in the case of smartglasses, this is not always the case, as the person experimenting with the marker naturally moves their head and has to consciously bring the visual marker into the camera’s field of view for the system to be able to track it.

In a first usability study, the new sensor system presented here combined with a Microsoft HoloLens 2 achieved a very good usability rating. In this study, a system usability score of 94.13 was achieved, which can be described as the “best imaginable”, following the classification by Bangor et al. [61]. The high usability score was also matched by a low rating regarding the interference by the HoloLens 2 during the experiment process, as rated by the participants. Compared with the first generation of sensors only containing the voltage and current measurement sensors, a higher score can be observed for the new system. Thees et al. [58] combined the previous sensor generation with a first-generation Microsoft HoloLens as well as an Apple iPad in a study evaluating the learning gain and cognitive load of students during the experiment. While both implementations showed good usability, the Apple iPad implementation achieved a higher usability rating. Nevertheless, the new system presented here achieved an even higher usability rating than the previous tablet implementation. This evidence for high usability and low interference of the new system could thereby enable further research targeting the use of AR—e.g., in regard to cognitive load and learning gain—and substantiate their results. However, both scores used in our study were at the upper or lower end of the used scales, which could lead to the ceiling or bottom effects limiting the validity of these results. Furthermore, the sample was limited to 20 participants. Analyzing the internal consistency of the scales, a low consistency was identified with values of Cronbach’s alpha slightly above 0.5 for the interference scale and 0.43 for the system usability scale. Traditionally, consistency values below 0.5 are deemed unacceptable, with values above 0.7 being seen as acceptable [66]. Previous use of the scale, however, showed acceptable consistencies, with the system usability scale also being an established scale to measure usability [57,58]. The low internal consistencies of both scales in this study could be the result of the aforementioned ceiling/bottom effects. As it is unclear if the scales still produce valid results with such high or low scores, the validity and consistency of the scales as well as the results should be reviewed in further studies.

However, the new system was only evaluated in a study exclusively focusing on the usability, and no full comparison including statistical tests was conducted. While the students conducted the same experimental procedure as the previous study targeting learning gain and cognitive load [58], no questionnaires regarding these variables were used, and the students were made aware that the focus of the study was the usability of the system and not their learning gain or cognitive load. This could have influenced the use of the system and its perceived usability. Furthermore, the sample from Thees et al. [58] consisted of university students, while this study was conducted at a school with voluntary participants and small sample size. These limitations could also affect the results and limits their transferability. Therefore, further evaluation of the usability in a study with students while also targeting the learning gain and cognitive load should be conducted to confirm these results.

Overall, the presented system provided seamless integration of multiple sensors into a real-world experiment setup, as described by Specht et al. [25]. This enabled the development of a new AR learning environment which was able to integrate MERs [38] in the form of the visualization of measurement values and the constructed electrical circuit. It also fulfilled the principles of spatial and temporal contiguity [47] as well as addressing the split-attention effect [52] by spatially integrating these virtualizations into the real environment and visualizing them in real-time. The high usability rating identified in the usability study also fulfills the need for highly usable AR environments, which are required for deeper investigations regarding the use of AR in learning settings [62]. While the system provides valuable possibilities and additions to a traditional experiment setup, its use needs to be reviewed carefully in the context of the learning task at hand. While the use of such a system has the potential to provide benefits while studying for example the laws in serial and parallel circuits, it also removes the handling of measurement devices such as multimeters from the experiment. The workings and correct use of such measurement devices should therefore be known to the student before starting to work on more complex tasks under the support of integrated measurement devices such as those presented here.

### 6.2. Further Development

There are currently multiple further developments planned to extend the sensor system presented in this work. Most of those developments are already in a prototype state, promising quick iteration.

While the voltage and current measurement approach integrated into our sensor boxes is targeted and optimized for DC experiments, future use cases will be extended to AC experiments, as well as transient responses of complex impedances. Such experiments require a higher accuracy regarding the current measurement in the lower measurement range, which cannot be provided sufficiently by the existent setup. Therefore, we are currently working on a measurement unit that incorporates multiple measurement ranges. In this way, a precise measurement for the lower measurement range is achieved without affecting the overall measurement range of the sensor box. We specifically focus on a mechanism that enables an uninterruptible switching between ranges to minimize the impact of the sensor box on the experiments. This will be combined with an increased data rate for an AC experiment enabling AC experiments with low frequencies.

These future developments will be combined with additional configuration options for the data packets sent by the sensor, e.g., configuring data rates and, if necessary, pre-filtering such as the smoothing of the sensor data. In this way, the sensors can be adapted according to the requirements of the experiment. Using these configuration options also enables the compatibility of our sensors with other already well-established apps such as Phyphox (https://phyphox.org, accessed on 28 October 2021). If a slow data rate is active, the processing is limited to converting the received measurement values from the received bytes into a numerical value by the processing application, followed by the offset and decimal place correction. After this simple conversion, it can immediately be used as the current measurement value.

While the current cable detection is limited to five plugs connected to each socket, the number of connected plugs can easily be extended if needed. However, increasing the number of plugs also reduces the response time due to the additional time required for checking the additional plugs. In the future, the integrated 1024 bit memory of the DS2431 will be used to store other information regarding the plug, such as the color and length of the cable, as well as the ID of the second plug. This additional information will eliminate the need to keep a static list of cables with their associated plug-IDs in the software and enable the use of previously unknown cables in an experiment without configuration changes.

**Figure 11:**
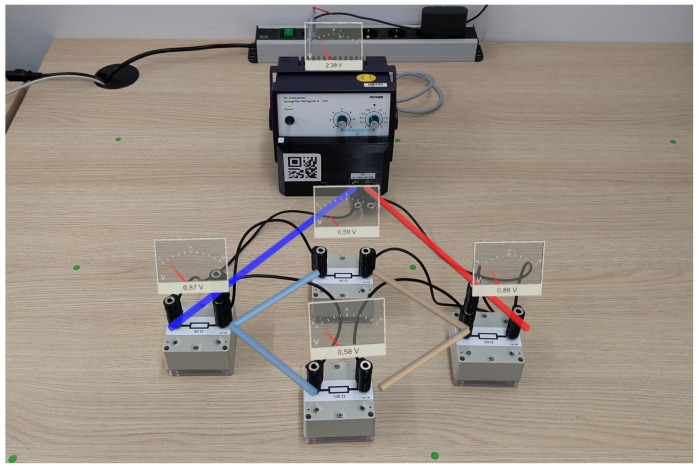
Example of a visualization of the electrical potential in the current circuit inspired by the electron gas model by Burde and Wilhelm [67].

Following the circuit reconstruction presented in this work and the validity check of the current circuit used in the presented usability study, additional uses of cable detection are planned. One use consists of the visualization of the potential and current through the circuit. Such visualizations could most importantly provide a connection between a theoretical discussion of electricity using models and real-world experiments (see Figure 11). Additionally, the circuit recognition method presented here could enable the real-time reconstruction of a circuit diagram matching the current setup. This could also help to provide a connection between a given circuit diagram to be constructed and the actual setup of the experiment while providing important insights into the currently constructed circuit in a more accessible format to identify the mistakes and issues of a complex circuit.

Even if, for the current experiment setup, a position detection in 2D is sufficient, a 3D localization is also possible. This could be achieved using a similar approach to the initialization of the 3D position of the base station. However, stable 3D position recognition likely requires more sensors and a 3D sensor placement instead of the current arrangement of all sensors on a 2D plane. The use of the positioning system in other experiments is also possible. For example, it could be used in the field of optics to track lenses on an optical table. Furthermore, the possibility of hand or finger tracking is also being considered.

All data generated by our sensor system as well as parts of the sensors themselves can also be used in other applications than the use case presented in our usability evaluation. Most importantly, the current sensor system provides a full digital representation of the experiment (digital twin). This enables new analysis and support options for the experiment based on the current setup. Such analysis and support could also be implemented independently from the visualization of measurement values, e.g., without using AR. One example could be individual feedback to each participant offering support when needed, as previously implemented using eye-tracking [68]. In addition, further learning analysis using AI can be conducted using the acquired sensor data, and additional sensor input such as mobile eye tracking in AR [69] can be used to further enhance the abilities of the presented sensor system and applications.

## 7. Conclusions

We have presented a new sensor system for STEM experiments covering basic electrical circuits in education settings. The system consists of custom sensor boxes and cables combined with an application for the Microsoft HoloLens 2 presenting the sensor data in an AR environment. Besides the electrical component used to construct the basic electrical circuits, our sensor boxes contain three different sensors. The first sensor measures the voltage across and current through the integrated component and reports the measurement data to the visualization application, which presents the values to the learner in an AR environment. The second sensor offers cable detection and recognizes the cables currently connected to the sensor box. These data are also reported to the application and are used to reconstruct the currently constructed circuit. The last sensor is used to locate the sensor box on the experiment table. The raw sensor data are thereby sent to the application, which calculates the box position and visualizes the measurement data spatially coherent to the sensor box.

The system reached a very high system usability score in an initial usability evaluation with n=20 pupils. Its use can therefore be recommended for further studies. This will enable future research into AR-supported learning and cognitive load research in AR while offering many possibilities for more sophisticated experiments and dynamic learner feedback and support including the use of AI for learning analysis.

## Figures and Tables

**Table 1:** Standard deviation of the position and rotation of the sensor box during the precision evaluation.

	x Position			z Position			y Rotation		
	SD in cm			SD in cm			SD in deg		
**Position**	**−50 cm**	**0 cm**	**50 cm**	**−50 cm**	**0 cm**	**50 cm**	**−50 cm**	**0 cm**	**50 cm**
30 cm	0.011	0.011	0.006	0.014	0.014	0.010	0.127	0.110	0.137
0 cm	0.011	0.003	0.012	0.013	0.006	0.012	0.080	0.076	0.198
−30 cm	0.008	0.007	0.009	0.011	0.017	0.010	0.128	0.102	0.106

**Table 2:** Standard deviation of the calculated position on the LFSR sequence for the two light-beams.

	1. Light Beam			2. Light Beam		
	SD in bit			SD in bit		
**Position**	**−50 cm**	**0 cm**	**50 cm**	**−50 cm**	**0 cm**	**50 cm**
30 cm	1.193	1.503	1.810	1.279	1.369	1.501
0 cm	1.051	1.394	1.310	1.130	1.593	1.318
−30 cm	0.950	1.269	1.060	1.211	1.057	1.196

**Table 3:** The adapted items of the interference scale originally developed by Kapp et al. [57] (translated for this publication).

Item	Text
1	I felt disturbed/impaired by the HoloLens.
2	would have performed better without the HoloLens.
3	I felt uncomfortable due to the HoloLens.

## Data Availability

The data presented in this study are available on request from the corresponding author.
